# Silent or Vocalizing Rats Copulate in a Similar Manner

**DOI:** 10.1371/journal.pone.0144164

**Published:** 2015-12-03

**Authors:** Anders Ågmo, Eelke M. S. Snoeren

**Affiliations:** Department of Psychology, University of Tromsø, Tromsø, Troms, Norway; University of Missouri, UNITED STATES

## Abstract

Both male and female rats produce 50 kHz ultrasonic vocalizations (USVs) in the presence of a sexual partner and during copulation. Previous studies showed that USVs have no incentive value for rats. In this study, we evaluated the role of USVs in behavior during copulation. Three groups of rats were used: sham males paired with sham females, devocalized females paired with sham males, and sham females paired with devocalized males. During the copulation test, the USVs emitted by the sham rat were recorded and the sexual behavior of both the male and the female were observed. The results revealed that devocalized and sham females showed similar patterns of sexual behavior and no difference was found in the copulatory behavior of devocalized and sham males. Also the behavior of the partner of a sham rat was comparable to the partner of a devocalized rat. In addition, almost no changes in USVs emission were found in the 5 seconds before and/or after a copulatory behavior. It can be concluded that USVs play no important role in rat copulatory behavior at least in sexually naïve rats.

## Introduction

It is often assumed that ultrasonic vocalizations (USVs) are important, as these sounds are emitted in several behavioral contexts, like during sexual behavior, rough and tumble play and aggressive interactions [1–3]. In a social context, it has been shown that rats approach male or female 50 kHz USVs in a radial maze [4, 5]. The 50 kHz USV stimuli used in these studies were recorded from rats during the exploration of a cage containing scents of a same-sex cage mate. It was therefore suggested that USVs have a communicative functions in rats [6, 7]. However, we have conducted several experiments in our laboratory that showed that male and female rats do not approach the playback of 50 kHz USVs emitted by the opposite sexes in a sexual behavior context [8, 9]. In addition, it was found that males and females approach devocalized females and males, respectively, in the same amount as vocalizing rats [8–10]. USVs also appear to be unimportant in a mate choice context, since female rats visited and copulated with silent, devocalized males as much as with vocalizing males [11]. These observations suggest that USVs do not serve a communicative function before or during sexual behavior. The fact that rats produce vocalizations in different situations does not necessarily mean that they have any consequences. While, our previous studies show that USVs do not attract conspecifics, it is still possible that USVs affect some aspects of sexual behavior during copulation.

In this study, we were interested in the role of USVs during copulation. Both male and female rats produce 50 kHz USVs in the anticipation to the introduction of a sexual partner, in the presence of a sexual partner and during copulation [7, 12–14]. It was found that the number of calls increases just before mounts and intromissions [15].

Studies with devocalized rats made it possible to investigate the role of USVs during copulation. Most studies performed in this field have used devocalized males, but the results are rather inconclusive. Several studies suggested that the emission of USVs by male rats induces paracopulatory behavior in females [10, 16]. Other studies, however, could not find a consistent effect of the emission of male USVs on paracopulatory behaviors [17], just as no effects of USV emission were found on lordosis responses [10, 16, 17]. The effects of male rats’ USV emission on male rat behavior is less investigated, but it was found that devocalized males show similar patterns of copulatory behavior as vocalizing males [16, 17], suggesting that the vocalizations produced by the male during mating are not self-stimulatory.

The role of USV emitted by female rats in sexual behavior, on the other hand, is not extendedly investigated. Only two studies have used devocalized females to investigate the role of USVs during copulation. In these studies it was shown that silent females only darted more than vocalizing females [18, 19], an effect that was partially reduced to control levels with the playback of USVs during the test. No other female behavior was affected consistently across the experiments. In addition, the emission of USV by females did not affect any parameter of male copulatory behavior [18].

Because the precise role of USVs during copulation is still inconclusive, this study investigated the effect of devocalization in either male or female rats on sexual behavior. If USVs play a communicative role during copulation, it can be expected that the behavior of the opposite sex will be affected by mating with a silent partner compared to vocalizing rats. On the other hand, if USVs play a self-stimulatory role, the behavior of the silent rats themselves should be different from the vocalizing rats. In addition, we recorded the USVs emitted by the intact rats during the sexual interaction. Hereby, we were able to investigate correlations between the emissions of certain types of USV calls and the behavior displayed.

## Methods

### Subjects

Thirty female and thirty male Wistar rats (250–300 grams at the start of the experiments) were obtained from Charles River (Sulzfeld, Germany). The rats were housed in same sex pairs in Macrolon IV^®^ cages on a reversed 12 hours light/dark cycle (light on between 11 pm and 11 am), in a room with controlled temperature (21±1°C) and relative humidity (55±10%). Standard rodent food and tap water were available ad libitum.

Ten females and ten males were devocalized under isoflurane anesthesia two weeks before the experiments. A 2-cm incision on the ventral surface of the neck was made, followed by the separation of the sternohyoideus muscles to expose the trachea and locate the recurrent laryngeal nerves. The nerve was freed from the surrounding fascia, lifted up and a section of about 3 mm of the nerve was removed bilaterally. The incision was closed with subcutaneous sutures. In addition, twenty males and twenty females received a sham treatment, in which the same procedure as the devocalization was followed, except for the section of the nerve. Buprenorphine (0.05 mg/kg s.c.) was administered to the rats at surgery and again every 12 hours for the following 3 days.

All thirty females were ovariectomized during the same isoflurane anaesthesia. Estradiol (EB) and progesterone (P) (Sigma, St. Louis, MO, USA) were dissolved in peanut oil (apoteksproduksjon, Oslo, Norway) and were given to the females subcutaneously in a volume of 0.2 ml/rat. The females received 18 μg/kg EB and 1 mg/kg P approximately 48 hours and 4 hours, respectively, before the start of the copulation test.

Before the copulation test, the sham and devocalized males and females were tested for the presence or absence of ultrasonic vocalizations (respectively) in the copulation cage. Unfortunately, one devocalized female and three devocalized males emitted USVs during this test, suggesting that the surgery was not successful. These rats were eliminated from the experiment. All the other devocalized rats were silent during the test and therefore considered successfully operated. The devocalized males did not emit 22-kHz USVs after ejaculation. The sham rats, on the other hand, produced as expected all subtypes of USVs.

### Behavioral procedure

#### Apparatus

The experiments were conducted in a round copulation cage with a diameter of 50 cm. The wall of the cage consisted of metal sheet covered with a black plastic surface. Sound absorbing isolation material of extruded polyethylene foam was used as cover for the inside of the cage. A high frequencies sensible microphone (obtained from Metris, Hoofddorp, The Netherlands) was placed above the cage adjusted so that all sounds from within the cage were registered. The microphone was connected to a computer with the Sonotrack^®^ sound analysis system. In addition, a video camera located above the cage was used to record the copulation test on video. Event recording software Observer XT 11 (obtained from Noldus, Wageningen, The Netherlands) was used to score the rat behavior during the copulation test. The tests were conducted under illumination of dim lights, which resulted in approximately 5 lux at the bottom of the cage.

#### Copulation test

All rats were sexually naive at the start of the experiment. One male and one female rat were placed in the copulation cage. They were allowed to copulate until the first postejaculatory intromission. If no ejaculation was reached, the test was terminated 20 minutes after the first intromission. If the male rat failed to perform an intromission within 20 minutes, the test was stopped.

Both, the ultrasonic vocalizations and the sexual behaviors were recorded and analyzed. The following parameters of sexual behaviors were observed in the female rats: the number of paracopulatory behaviors (darts and hops), the lordosis quotient (lordosis responses/(mounts+intromissions+ejaculation)*100), the number of extra lordoses (lordosis responses without a mount, intromission or ejaculation), and the latency to the 1^st^ paracopulatory behavior (time to the first dart or hop). In the males, the number of mounts, intromissions and ejaculation, the mount latency (time to the first mount with pelvic thrusting), intromission latency (time to the first mount with vaginal penetration), and the ejaculation latency (time from first intromission to ejaculation) were measured. In addition, we calculated the intromission ratio (number of intromissions/(number of intromissions+number of mounts) and the interintromission interval (time from first intromission to ejaculation (or last copulatory event if the male did not achieve ejaculation)/number of intromissions).

### Design

The rats were divided in three groups: sham females paired with sham males (n = 10), sham females paired with devocalized males (n = 7), and devocalized females (n = 9) paired with sham males.

### USV analysis

After the completion of the experiments, the spectrograms of the recorded USVs were analyzed manually by a trained observer. The following USV subtypes were distinguished: complex, step down, upward ramp, multistep, downward ramp, trill, flat, flat-trill combination, short, trill with jumps, inverted-u, step up, composite and 22 kHz calls. The characteristics of the different types of USVs are described in Wright et al. (2010) [20]. The USVs emitted by the sham females in group sham females-devocalized males and the sham males in the group devocalized females-sham males (see Design) were analyzed. The USVs in the group sham females-sham males were not analyzed, since both rats were sham operated and so it was impossible to determine whether the USVs were emitted by the male or the female. Five seconds before and after each paracopulatory behavior, lordosis response, mount or intromission, the USVs were analyzed. The USVs emitted before and after an ejaculation were not analyzed, since there was maximum one ejaculation per test and only 2 males per group ejaculated.

Insofar, the reason for emitting USVs by rats is unknown. It is therefore impossible to determine whether the calls are produced in relationship to their own behaviors or in response to the partner’s behavior. In addition, it is unclear whether the USVs are emitted before or after the behaviors. Therefore, we have analyzed the data from all perspectives, meaning that we first analyzed the data by counting the USVs emitted 5 seconds before each female behavior, followed by analyzing the same data by counting the USVs emitted before each male behavior. In case the rat performed two behaviors within the 5 seconds, the USVs were only counted for the first behavior. This was done again for the USVs emitted 5 seconds after each female and male behavior, but this time the USVs were only counted for the last behavior when more behaviors took place within the 5 seconds.

### Data analysis

The Shapiro-Wilk test was used to determine whether the data were normally distributed. Most of the parameters were not normally distributed. Therefore, all parameters were analyzed with the non-parametric Kruskal Wallis test when the three groups were compared. Since only 7 males (n = 2 for sham females paired with devocalized males, n = 2 for devocalized female paired with sham males, and n = 3 for sham females paired with sham males) achieved an ejaculation during this experiment, proper statistical comparisons cannot be made. Therefore, we excluded the number of ejaculations and the latency to ejaculation from the data analysis.

For the ultrasonic vocalization data, we investigated whether or not there were significantly more or less USVs emitted during the 5 seconds before or after the behaviors compared to when the vocalizations would have been emitted in an equally distributed manner over the time. Therefore, we calculated for each rat first the number of USVs for the “equally distributed USVs” by dividing the total number of USVs emitted during the test by the test duration in seconds. The result was then multiplied by 5 in order to obtain the number of calls that would have been emitted during 5 seconds if calls were randomly distributed during the test. In addition, we calculated the “actual emitted” number of USVs for each rat during the 5 seconds before and after a paracopulatory behavior, lordosis, mount or intromission. For this, the average number of emitted calls before each of these behavioral event in each animal was calculated. For instance if one male mounted 6 times, we determined the total number of emitted calls before these 6 mounts and then divided that number by 6. Then we determined the “fraction of actual emitted USVs” by calculating the averages of “actual emitted USVs” minus the “equally distributed” USVs. In this way we controlled for the interindividual differences in the number of copulatory events. The “fraction of actual emitted USVs” were compared to 0 with Wilcoxon signed rank test. Since multiple tests are used for testing our hypothesis, we employ the Bonferroni correction in order to protect significance levels.

### Ethics

All experimentation was conducted in agreement with the European Union council directive 86/609/EEC and approved by the National Animal Research Authority in Norway.

## Results

### Sexual behavior

**Fig 1:**
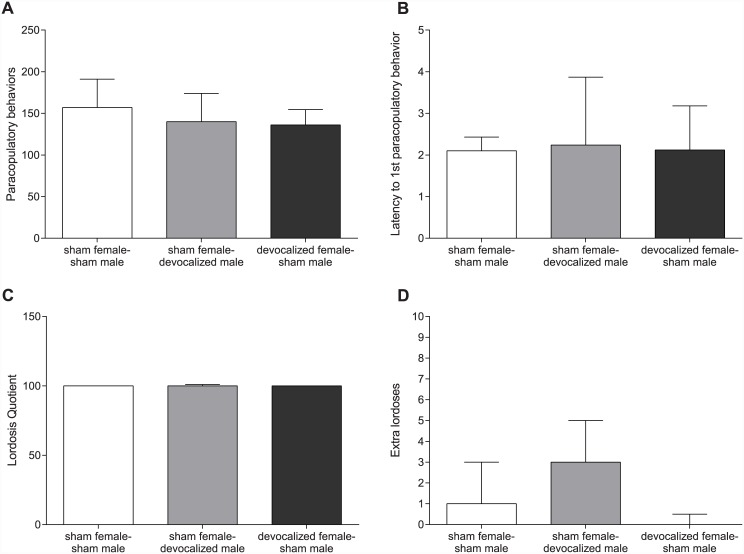
No differences in female copulatory behavior. (A) The number of paracopulatory behaviors, (B) the latency to the first paracopulatory behavior, (C) the lordosis quotient, and (D) the number of extra lordoses performed by the females. All graphs show the comparison between sham females paired with sham males, sham females paired with devocalized males and devocalized females paired with sham males. Data are shown as median ± semi interquartile range.

Analysis of the data revealed that there were no significant differences in the number of paracopulatory behaviors, the latency to the 1^st^ paracopulatory behavior, the lordosis quotient or the number of lordosis responses in the absence of a mount or intromission in the female sexual behavioral parameters between the groups with sham females-sham males, sham females-devocalized males, and devocalized females-sham males (Fig 1).

**Fig 2:**
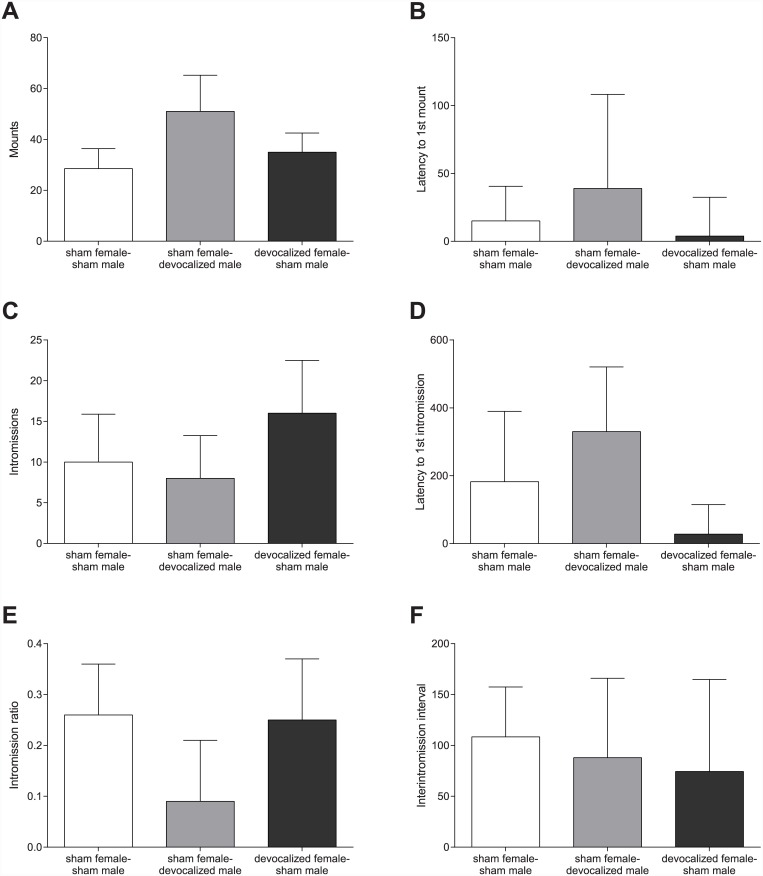
No differences in male copulatory behavior. (A) The number of mounts, (B) the latency to the first mount, (C) the number of intromissions, (D) the latency to the first intromission, (E) the intromission ratio, and (F) the interintromission interval performed by the males. All graphs show the comparison between sham females paired with sham males, sham females paired with devocalized males and devocalized females paired with sham males. Data are shown as median ± semi interquartile range.

In addition, no significant differences were found in the male sexual behavior parameters between the groups with sham females-sham males, sham females-devocalized males, and devocalized females-sham males: a similar number of mounts and intromissions were performed by the sham and devocalized males or received by the sham or devocalized females. Devocalized males showed the same latency to the first mount or intromission, intromission ratio, and interintromission interval as the sham males (Fig 2).

### Female ultrasonic vocalizations and sexual behavior

**Fig 3:**
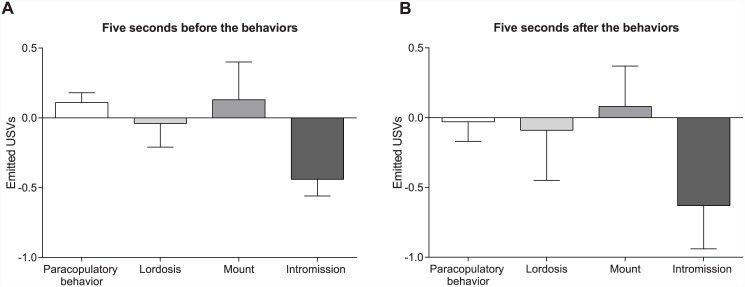
No changes in emission of female USVs before and after a behavior. (A) The average number of ultrasonic vocalizations (USVs) emitted by female rats (paired with devocalized males) during the five seconds before, and (B) the five seconds after a paracopulatory behavior, lordosis, mount, or intromission. Both graphs show the comparison between USVs emitted before or after the behaviors minus the USVs that would have been emitted during 5 seconds in the equally distributed USVs. Data are shown as median ± semi interquartile range.

The analysis of the data concerning the USVs emitted by sham females revealed that there was neither an increase nor a decrease in number of emitted USVs during the 5 seconds before (Fig 3A) and after (Fig 3B) a paracopulatory, lordosis, mount or intromission compared to the emitted USVs based on the “equally distributed USVs”.

### Male ultrasonic vocalizations and sexual behavior

**Fig 4:**
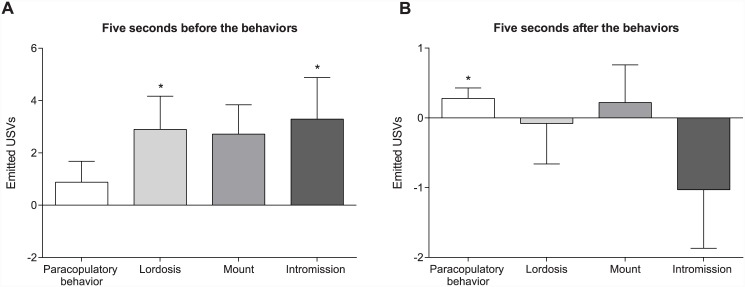
Increased emission of male USVs before an intromission or lordosis. (A) The average number of ultrasonic vocalizations (USVs) emitted by male rats (paired with devocalized females) during the five seconds before, and (B) the five seconds after a mount, intromission, lordosis, or paracopulatory behavior. Both graphs show the comparison between USVs emitted before or after the behaviors minus the USVs that would have been emitted during 5 seconds in the equally distributed USVs (*p<0.05 compared to zero). Data are shown as median ± semi interquartile range.

In the sessions containing a sham male and a devocalized female, Wilcoxons analysis showed a significant difference compared to zero for the “fraction of actually emitted USVs” during the 5 seconds before an intromission (Z = -2.547, p = 0.011; Fig 4A), 5 seconds before a lordosis response (Z = 2.547, p = 0.011; Fig 4A), and 5 seconds after a paracopulatory behavior (Z = -2.666, p = 0 008; Fig 4B).

Since the male rats emitted more USVs during the 5 seconds before an intromission or lordosis, and 5 seconds after a paracopulatory behavior, these USVs were divided into the different subtypes of USVs as described in Wright et al. (2010) and statistically analyzed again. The Wilcoxons analysis revealed significant differences of the “fraction of actually emitted USVs” compared to zero in complex calls (Z = -2.547, p = 0.011) during the 5 seconds before an intromission, and in upward ramp calls during the 5 seconds before a mount (Z = -2.547, p = 0.011) and lordosis (Z = -2.547, p = 0.011) (Table 1).

**Table 1:** Only a few changes in emission of male USV subtypes before and after a behavior.

**A: USV subtype**	**Before paracopulatory behavior**	**Before lordosis**	**Before mounts**	**Before intromissions**
Complex	0.10±0.24	0.36±0.35	0.45±0.37	0.36±0.26*
Flat	0.06±0.04	0.17±0.11	0.11±0.12	0.08±0.16
Trill	0.26±0.37	1.21±0.61	1.18±0.56	1.38±0.61
Inverted-U	0.01±0.02	0.03±0.02	0.02±0.05	0.00±0.08
Upward ramp	0.12±0.11	0.25±0.29*	0.17±0.28*	0.41±0.40
Downward ramp	0.00±0.01	0.02±0.01	0.01±0.03	0.00±0.10
Step up	0.00±0.01	0.02±0.02	0.00±0.01	0.00±0.02
Step down	0.01±0.01	0.02±0.01	0.02±0.01	0.00±0.00
Short	0.03±0.01	0.01±0.03	0.05±0.03	-0.01±0.02
Flat-trill combination	0.02±0.03	0.04±0.05	0.06±0.06	0.00±0.11
Multistep	0.03±0.02	0.06±0.06	0.03±0.05	0.17±0.13
Trill with jumps	0.00±0.00	0.00±0.00	0.00±0.01	0.00±0.02
22-kHz	0.00±0.00	0.00±0.00	0.00±0.00	0.00±0.00
Composite	0.00±0.00	0.00±0.00	0.00±0.00	0.00±0.00
**B: USV subtype**	**After paracopulatory behavior**	**After lordosis**	**After mounts**	**After intromissions**
Complex	0.15±0.26	0.05±0.42	-0.03±0.05	-0.11±0.14
Flat	0.05±0.03	-0.08±0.0	-0.01±0.10	-0.09±0.07
Trill	0.08±0.06	-0.10±0.14	0.01±0.18	-0.33±0.19
Inverted-U	0.01±0.04	0.00±0.06	-0.01±0.01	-0.02±0.01
Upward ramp	0.01±0.04	-0.04±0.04	-0.01±0.10	-0.10±0.09
Downward ramp	0.02±0.05	0.01±0.02	0.00±0.02	-0.01±0.00
Step up	0.00±0.01	0.00±0.02	0.00±0.01	0.00±0.01
Step down	0.01±0.01	0.00±0.00	0.00±0.00	-0.01±0.00
Short	0.00±0.00	0.00±0.02	-0.01±0.01	-0.02±0.01
Flat-trill combination	0.00±0.01	0.00±0.02	0.00±0.00	-0.01±0.02
Multistep	0.01±0.01	0.00±0.01	0.00±0.02	-0.04±0.02
Trill with jumps	0.00±0.01	0.00±0.00	0.00±0.00	-0.01±0.01
22-kHz	0.00±0.00	0.00±0.00	0.00±0.00	0.00±0.00
Composite	0.00±0.00	0.00±0.00	0.00±0.00	0.00±0.00

(A) The average number of ultrasonic vocalization (USV) subtypes emitted by male rats (paired with devocalized females) during the five seconds before, and (B) the five seconds after a paracopulatory behavior, lordosis, mount, or intromission. Both graphs show the comparison between the USV subtypes emitted before or after the behaviors minus the USV subtypes that would have been emitted during 5 seconds in the equally distributed USVs. Data are shown as median ± semi interquartile range.

*p<005 compared to zero.

## Discussion

The present experiment showed that devocalization did not affect copulatory behaviors in sexually naïve male and female rats. Pairs of vocalizing rats (sham female-sham male) showed a similar pattern of sexual behavior as pairs in which either the male (sham female- devocalized male) or the female (devocalized female-sham male) was devocalized. This suggests that ultrasonic vocalizations do not play an important role during copulation. The results were in line with other studies in which no effects on sexual activity were found when the males or females were silent [16–18, 21]. The number of mounts [16, 17, 21], intromissions [16, 17, 21] and paracopulatory behaviors [17, 21] remained unaffected when males were devocalized. Devocalization of females or deafening of males did also not consistently affect mounts, intromissions or lordosis behavior [18].

However, in the studies by White et al. (1990), it was found that females were less likely to remain stationary when devocalized males attempted to mount. Instead they move away during mounting before the male could intromit [17, 21]. Although, the total number of intromissions was not affected [21], it was suggested that USVs affect the coordination of sexual behavior between males and females. Fig 2 of our study shows an insignificant trend of reduction in the latency to 1^st^ intromission in the group with devocalized females paired with sham males, and a reduction in intromission ratio for the sham females paired with devocalized males. This could also indicate the involvement of USVs in the coordination of sexual behavior between males and females. However, this effect is not significant because the variation between rats is very large. Therefore, it is impossible to conclude that USVs affect the coordination of sexual behavior between males and females. The wide variation in latency parameters between animals is normal for sexually naive rats [22]. Our experiment showed that the emission of USV had no effect on the intromission latency or intromission ratio. Therefore, we propose that ultrasonic vocalizations are not important in copulatory behavior.

One might suggest that it would have been better to use sexually experienced rats instead of inexperienced. However, if USVs were important for the coordination of sexual behavior between males and females, it would be especially important during their first sexual interaction. As shown in experiments with anosmic rats [23], rats are mainly affected by missing cues during their first sexual experience. Later on they develop strategies to compensate for the information that is lacking. Thus the role of USVs in sexual interaction should be most evident during their first sexual encounter. The use of inexperienced subjects thus maximized the likelihood of finding effects of USVs However, based on our data we have to conclude that USVs play a minor or no role.

In addition, our study contradicts some other studies in which female rats tend to dart less when paired with a devocalized male [10, 16]. However, the study by Thomas et al. also revealed that devocalization has no effect on the choice of male (vocalizing or silent) in terms of number of visits or on the time spent with each male [10]. Our data is also in conflict with studies in which the females were devocalized and showed that silent females dart more than sham females [18, 19]. Interestingly, vocalizing females also showed more paracopulatory behaviors towards deafened males compared to intact males in the same study [18]. This suggests that the increase in paracopulatory behaviors is not a result of the lack of female USVs, but could just be caused by the male’s behavior. It was previously shown that the playback of USVs actually does not induce the display of paracopulatory behaviors [24].

The absence of USVs did not affect copulatory behavior in our experiment. This suggests that USVs do not have a communicative function during copulation in sexually naïve rats. If USVs would communicate something, the behavior should have been different in silent rats compared to vocalizing rats. The finding that USVs are not involved in the regulation of behavior was also found in play behavior. It was shown that juvenile rats emit more USVs before play events, but no differences in play behavior were found between devocalized and sham rats [25, 26]. In addition, silent rats were selected as often for play events as vocalizing rats [25, 26]. The only difference in behavior was found when both rats were devocalized; there were more attack like behaviors compared to sham [26]. All together these studies suggest, in line with our studies on sexual behavior, that emitting USVs is not essential for promoting a playful mood, locating a partner, attracting play partners or preventing play fights from escalating into serious fights when confronted with an ambiguous social context [26].

In our study, we also examined the link between emitted USVs and behavior. If USV are used to communicate something, it could be expected that certain types of vocalizations are emitted right before or after a particular behavior. Previously, it has been suggested that the emission of USVs increased in the seconds before a mount and intromission [1]. It was also suggested that more USVs were emitted before an intromission than before a mount. However, the rats used in this study were not devocalized, resulting in the recording of both male’s and female’s USVs. In our experiment only one of both rats was devocalized allowing recording the USVs emitted by either the male or female rat. In this experiment, we could accurately determine the emitter of USVs and thereby we obtained data far more reliable than those from the earlier studies.

Analysis of the results showed that there is no link between the emission of female USVs and the display of behavior. As shown in Fig 3, female rats did not emit more or less USVs the 5 seconds before a paracopulatory behavior, lordosis, mount or intromission. In other words, the emitted vocalizations (in number or type of call) before behaviors do not contain information about the behavior that is coming, otherwise there should have been a difference with zero. In addition, the behavior displayed did not induce the emission of USVs afterwards. Therefore, it can be concluded that female USVs have no direct significance for copulatory behavior neither in male, nor in female rats.

Interestingly, males actually did emit more USVs in the 5 second before an intromission or lordosis (Fig 4). After these behaviors, on the other hand, no differences in emitted USVs were found. However, the males emitted more USVs during the 5 seconds after a paracopulatory behavior, although this increase was rather limited. As mentioned before, it is unknown whether USVs are emitted to affect the behavior of the vocalizing rat or its partner, or for some other reason. The females in this experiment showed a lordosis quotient of 100%, implicating that all male behaviors were followed by a lordosis response. This means that in the female, the calls counted in the 5 seconds before (or after) a mount or intromission are the same calls as observed before the lordosis. Therefore, it is impossible to conclude whether the USVs are linked to the male behaviors or the lordosis. However, no link was found between the emission of USVs before and the display of paracopulatory behaviors. In addition, a 100% lordosis quotient is also reached without the presence of USVs, since silent males received as often lordosis responses as vocalizing males. Together, this suggests that the probability that males emit USVs in order to induce sexual behavior in females is rather low.

It should be mentioned though that even though the increase is rather small, the males emitted significantly more USVs after a paracopulatory behavior than expected from the equally distributed USVs. This effect could be easily explained by the fact that many of the paracopulatory behaviors are quickly followed by a mount or intromission of the male. If this happened within the 5 second interval, the USVs were double counted: in the “after paracopulatory” and in the “before mount or intromission” category. Since the males emitted more USVs before a intromission, it could be expected that some of these calls are also visible in the category after paracopulatory behaviors.

The possibility that males emit USV as a kind of self-regulatory stimulus is also unlikely. If male rats emit USVs in order to increase their own sexual activity, it could be expected that vocalizing males would copulate more actively than silent males. However, this is not supported by our data in which devocalized males have the same mating patterns as sham males. In addition, no link was found between the different types of USV and the types of behavior. Male rats did indeed emit more complex calls before intromissions, and more upward ramp calls before mounts, but the type of emitted call is no predictor of the behavior that is following, since complex and upward ramp calls are also emitted before other copulatory behaviors. Similar results have been found with regard to play behavior. Juvenile rats emit more calls before an attack, but there is no correlation to a certain type of USV [27]. Therefore, we conclude that although males emit more USVs in the 5 seconds before an intromission, these USVs play no important role during copulation in sexually naïve rats.

It seems that the emission of USVs is more an irrelevant by-product of sexual arousal. Similar to birds and humans, USVs in rats have been shown to be associated with increased subglottal pressure which is related to the breathing cycle [28–31]. As described in the review by Blumberg, ultrasounds can be linked to locomotion in many species including rats, and can therefore be a by-product of thoracic compression during locomotion [32]. The increased arousal before mounts and intromissions might increase the breathing and therefore generate USVs. This theory is in line with a statement by Robert W. Bell published in 1974: “*All of the circumstances which lead to the brief*, *high-frequency adult signals involve a high degree of arousal (fighting*, *copulation*, *being shocked)*, *whereas the 2* ^*nd*^ *type of adult signal seems to be correlated with an abrupt decrease from a high arousal state (following fighting*, *following ejaculation*, *following cessation of shock)*. *Thus*, *changes in acoustical parameters of the vocalizations may reflect simply the energy in emitting the signals*.” [33].

In conclusion, this experiment showed that vocalizing and silent rats copulate in a similar manner. There were no differences in behavior between devocalized and sham rats neither in males, nor in females. In addition, no relevant changes in USVs emission were found in the 5 seconds before and/or after a copulatory behavior, suggesting that USVs do not play a role (or mostly a minor role) in copulation. USVs can only have communicative properties if they would have behavioral consequences. Therefore, our experiments indicate that ultrasonic vocalizations have no communicative function during copulatory interaction at least in sexually naïve rats.
